# Case report: Strong GAD antibody positivity and type 1 diabetes-HLA-susceptible haplotype-DRB1*04:05-DQB1*04:01 in a Japanese patient with immune checkpoint inhibitor-induced type 1 diabetes

**DOI:** 10.3389/fendo.2024.1407192

**Published:** 2024-05-22

**Authors:** Shunya Yabuki, Hiroyuki Hirai, Chihiro Moriya, Yoshiro Kusano, Takeo Hasegawa

**Affiliations:** ^1^ Department of Third Internal Medicine, Shirakawa Kosei General Hospital, Shirakawa, Japan; ^2^ Department of Chest Surgery, Shirakawa Kosei General Hospital, Shirakawa, Japan

**Keywords:** immune checkpoint inhibitors, glutamic acid decarboxylase antibody, type 1 diabetes mellitus, human leukocyte antigen haplotype, Japanese

## Abstract

Immune checkpoint inhibitors (ICIs) are widely used in cancer treatment; however, they can lead to immune-related adverse events, including immune checkpoint inhibitor-induced type 1 diabetes mellitus (ICI-T1DM). While fulminant T1DM is common in East Asia, ICI-T1DM has predominantly been reported in Western countries. In this report, we present the case of a 66-year-old Japanese man with type 2 diabetes mellitus undergoing dialysis for diabetic nephropathy. The patient was diagnosed with left upper lobe lung cancer, and treatment with nivolumab and ipilimumab was initiated. After 48 days, the patient experienced impaired consciousness and difficulty moving. His blood glucose levels were 815 mg/dL, and metabolic acidosis was detected, leading to a diagnosis of diabetic ketoacidosis. The patient was subsequently treated with continuous intravenous insulin. However, his C-peptide levels rapidly depleted, and new-onset ICI-T1DM was diagnosed. Although most Japanese patients with ICI-T1DM test negative for glutamic acid decarboxylase (GAD) antibodies, this case exhibited a strong positivity. Thus, we reviewed the literature on 15 similar Japanese cases, revealing a mean HbA1c level at onset of 8.7% and a mean time from ICI administration to onset of 9.7 weeks, which was shorter than that in GAD-negative cases. Moreover, human leukocyte antigen typing revealed five cases of DRB1*04:05-DQB1*04:01, including the present case, and one case of DRB1*09:01-DQB1*03:03, both of which were susceptible to T1DM haplotypes. These findings suggest that GAD antibody positivity may be associated with acute onset and disease progression in some cases of Japanese patients with ICI-T1DM. Given that the prediction of new-onset ICI-T1DM is challenging, monitoring GAD antibody levels might be useful. However, further studies with large sample sizes and validation across different racial and ethnic populations are warranted.

## 1 Introduction

Immune checkpoint inhibitors (ICIs) have been widely used for the treatment of many cancers owing to their potent anti-tumor effects, achieved by inhibiting molecules such as cytotoxic T-lymphocyte antigen 4 (CTLA-4), programmed cell death 1 (PD-1), and programmed cell death ligand 1 (PD-L1) (1). However, immune-related adverse events following the use of ICIs have been increasingly reported (2, 3). Fulminant type 1 diabetes mellitus (T1DM) is prevalent in East Asia, including Japan (4). ICI-induced T1DM (ICI-T1DM) has been widely reported in many Western countries, and its onset resembles that of fulminant T1DM (5). ICI-T1DM and acute T1DM are similar yet different conditions classified as “checkpoint inhibitor-associated autoimmune diabetes mellitus (CIADM)” (6), and ICI-T1DM has been recognized as a new category within T1DM.

Glutamic acid decarboxylase (GAD) antibody and human leukocyte antigen (HLA) haplotypes are recognized for their roles in the diagnosis and pathogenesis of T1DM (7, 8). However, their roles in ICI-T1DM have not been yet determined. Baden et al. recently reported that the prevalence of GAD antibody positivity in ICI-T1DM is lower in Japan than in Western countries (9). Moreover, in Western countries, GAD antibody-positive ICI-T1DM has a more acute onset than GAD antibody-negative ICI-T1DM (5, 10–13). However, evidence from Japanese patients is lacking. Although it has been reported that DRB1*04:05-DQB1*04:01 and DRB1*09:01-DQB1*03:03 are common HLA haplotypes in Japanese patients with acute-onset and fulminant T1DM (14, 15), the association between HLA haplotypes and Japanese ICI-T1DM remains unclear.

In this report, we present a case of GAD antibody positivity and T1DM-susceptible haplotype-DRB1*04:05-DQB1*04:01 in ICI-T1DM and review GAD antibody positivity and HLA haplotypes in Japanese ICI-T1DM cases.

## 2 Case description

A 66-year-old man with a history of diabetes since 43 years of age had been under treatment with antidiabetic agents, including alpha-glucosidase inhibitors, sulfonylureas, and thiazolidinediones. However, owing to inadequate control, he required the administration of a subcutaneous injection of long-acting insulin. At 47 years of age, his serum C-peptide concentration was 0.9 ng/mL. At 59 years of age, he required hemodialysis for diabetic nephropathy. He was treated with 6 units of insulin glargine subcutaneously injected before bedtime and 0.3 mg of voglibose before each meal at another hospital. His fasting blood glucose ranged from 120 to 180 mg/dL, and his HbA1c levels ranged from 6.0 to 7.3%.

At 66 years of age, at our hospital, immunotherapy with nivolumab and ipilimumab was initiated for left upper lobe lung cancer (c-T2aN1M1a Stage 4A), with the initiation of immunotherapy defined as day 1. After starting immunotherapy, the patient experienced diarrhea and itching, which were considered side effects of immunotherapy. Accordingly, treatment with 10 mg/day of prednisolone was initiated concurrently with the third nivolumab administration on day 43.

**Figure 1:**
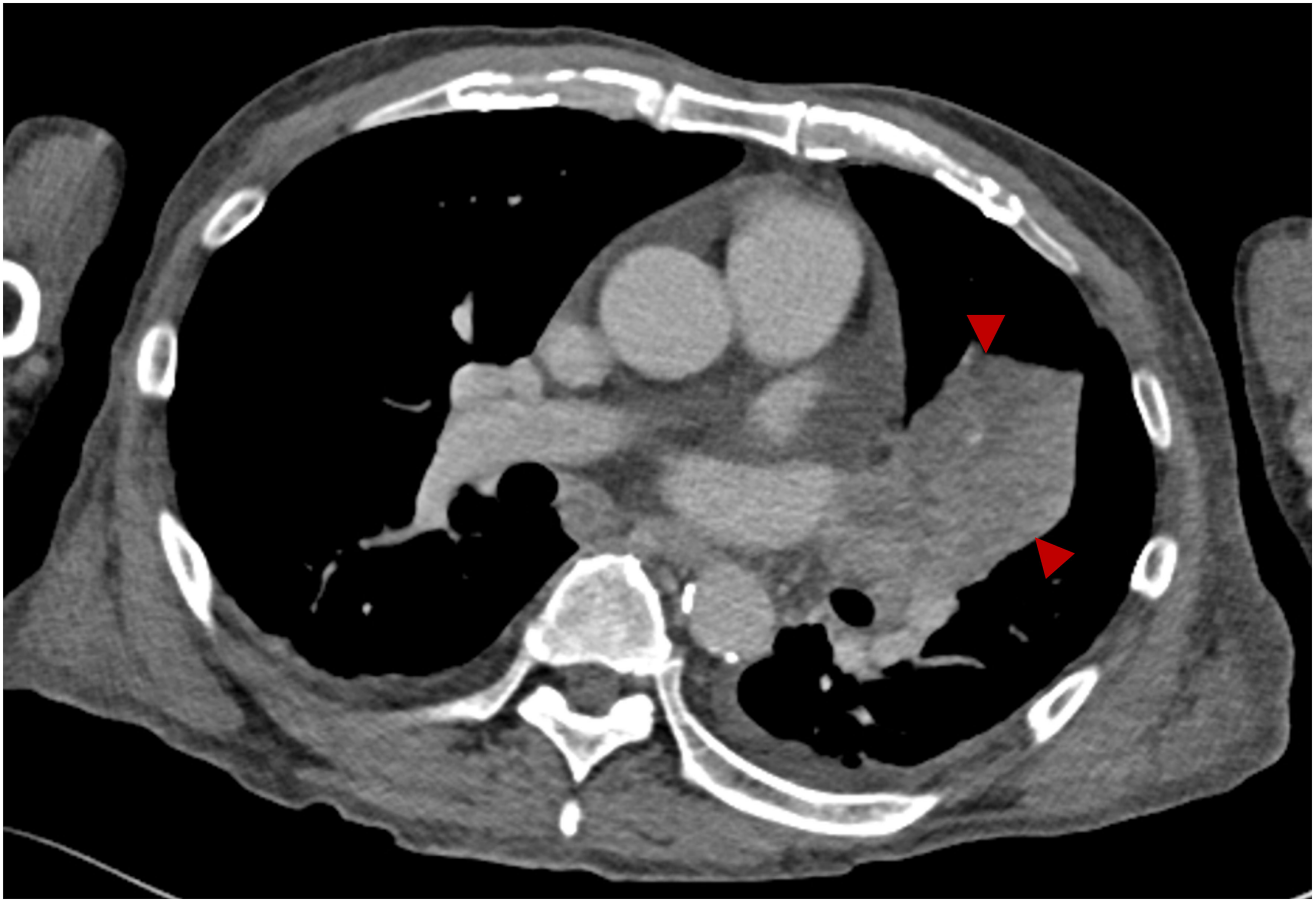
Contrast-enhanced computed tomography on admission. Contrast-enhanced computed tomography showing left upper lobe lung cancer (arrowheads).

On the fifth day of the third nivolumab administration (day 47), the patient began experiencing symptoms of fatigue, followed by difficulty moving the next morning, prompting hospitalization on that day. He had a history of hypertension and dyslipidemia but no relevant family history. His physical parameters were as follows: height, 164 cm; weight, 62.1 kg; body mass index, 23.1 kg/m^2^; blood pressure, 155/137 mmHg; pulse, 93/min; body temperature, 36.8°C; and oxygen saturation, 95% (ambient air). The patient was unconscious; however, no obvious paralysis or neck rigidity was observed. The blood test results are presented in **Table 1**. His blood glucose level was 815 mg/dL, with HbA1c elevated to 8.7%. Plasma osmolality was 318 mOsm/L, and ketosis and metabolic acidosis were noted. His serum C-peptide level was 0.42 ng/mL, and the GAD antibody level exceeded 2000 U/mL. Chest radiography and computed tomography (**Figure 1**) confirmed known lung cancer, with no additional findings. Based on these findings, the patient was clinically diagnosed with diabetic ketoacidosis (DKA).

**Table 1:** Patient laboratory data on admission.

	Value	Reference range		Value	Reference range
Blood cell count			Blood gas analysis (artery)		
RBC (×10^6^/µL)	4.81	4.35–5.55	pH	7.337	7.350–7.450
Hb (g/dL)	13.1	13.7–16.8	pO_2_ (mmHg)	56.7	83–108
WBC (×10^3^/µL)	6.4	3.3–8.6	pCO_2_ (mmHg)	35.2	35–48
Plt (×10^3^/µL)	225	158–348	HCO_3-_ (mEq/L)	18.4	21–28
Biochemistry			Lactate (mmol/L)	0.7	0.5–1.6
TP (g/dL)	7.9	6.6–8.1	Diabetes-related examination		
Alb (g/dL)	3.6	4.1–5.1	PG (mg/dL)	815	73–109
AST (U/L)	13	13–30	HbA1c (NGSP, %)	8.7	4.9–6.0
ALT (U/L)	13	10–42	C-peptide (ng/mL)	0.42	0.61–2.09
ALP (U/L)	95	38–113	Anti-GAD antibody (U/mL)	>2000	0.0–1.5
γ-GT (U/L)	13	13–64	Anti-IA-2 antibody (U/mL)	<0.6	0.0–0.6
LDH (U/L)	329	124–222	Anti-insulin antibody (U/mL)	<0.4	0.0–0.4
T-Bil (mg/dL)	0.31	0.40–1.50	Endocrinological examination		
NH_3_	39	30–86	ACTH	105.0	7.2–63.3
BUN (mg/dL)	44	8.0–20.0	Cortisol (μg/dL)	16.6	6.2–19.4
Cre (mg/dL)	8.00	0.65–1.07	TSH (µIU/mL)	3.24	0.35–4.94
CK (U/L)	95	59–248	FT3 (pg/mL)	<1.5	1.88–3.18
Na (mEq/L)	126	138–145	FT4 (ng/dL)	0.94	0.70–1.48
K (mEq/L)	5.4	3.6–4.8	TSH receptor antibody (IU/mL)	1.83	0.00–3.10
Cl (mEq/L)	92	101–108	Anti-TPO antibody (IU/L)	4.4	0.0–3.3
Ca (mg/dL)	8.7	8.8–10.1	Anti-Tg antibody (IU/L)	13.0	0.0–19.3
CRP (mg/dL)	0.20	0.00–0.14	HLA typing analysis		
Total ketone body	1314	26–122	A*11:01:01, 24:02:01		
3–hydroxybutyric acid	808	0–76	B*39:01:01, 54:01:01		
Acetoacetate	506	13–69	C*01:02:01, 07:02:01		
			DRB1*04:05:01, 13:02:01		
			DQB1*04:01:01, 06:04:01		
			DQA1*01:02:01, 03:03:01		

ACTH, adrenocorticotropic hormone; Alb, albumin; ALP, alkaline phosphatase; ALT, alanine aminotransferase; AST, aspartate aminotransferase; BUN, blood urea nitrogen; Ca, calcium; CK, creatine kinase; Cl, chloride; Cre, creatine; CRP, C–reactive protein; FT3, free triiodothyronine; FT4, free thyroxine; GAD, glutamic acid decarboxylase; γ–GT, γ–glutamyl transpeptidase; Hb, hemoglobin; HbA1c, glycated hemoglobin; HCO3–, bicarbonate; HLA, human leukocyte antigen; IA–2, insulinoma‐associated antigen–2; K, potassium; LDH, lactate dehydrogenase; Na, sodium; NGSP, National Glycohemoglobin Standardization Program; NH_3_, ammonia; pCO_2_, partial pressure of carbon dioxide; PG, plasma glucose; pH, potential hydrogen; Plt, platelets; pO_2_, partial pressure of oxygen; RBC, red blood cell; T-bil, total bilirubin; Tg, thyroglobulin; TP, total protein; TPO, thyroid peroxidase; TSH, thyroid stimulating hormone; WBC, white blood cell.

Upon admission, a central venous catheter was inserted. As the patient was on dialysis, the infusion was minimized, and treatment focused on a continuous intravenous insulin infusion, ranging from 0.2 to 5.0 U/h. By day 3, the patient’s consciousness had gradually improved. As his blood glucose level decreased and his metabolic acidosis improved, continuous intravenous insulin infusion was terminated, and subcutaneous insulin injection was initiated (14–25 U/day). However, as blood glucose levels fluctuated, insulin injection levels were finely adjusted. On the 17th day of hospitalization (day 64), the patient was finally discharged with multiple daily injections of insulin aspart 6–6-4 and insulin glargine 0–0-0–9. His serum C-peptide levels decreased to 0.26 ng/mL and <0.03 ng/mL on days 63 and 94, respectively. Given his medical history and highly positive anti-GAD antibody test results, we diagnosed the patient with autoimmune T1DM caused by ICIs. HLA typing identified DRB1*04:05-DQB1*04:01, which is reportedly associated with T1DM susceptibility (**Table 1**).

**Figure 2:**
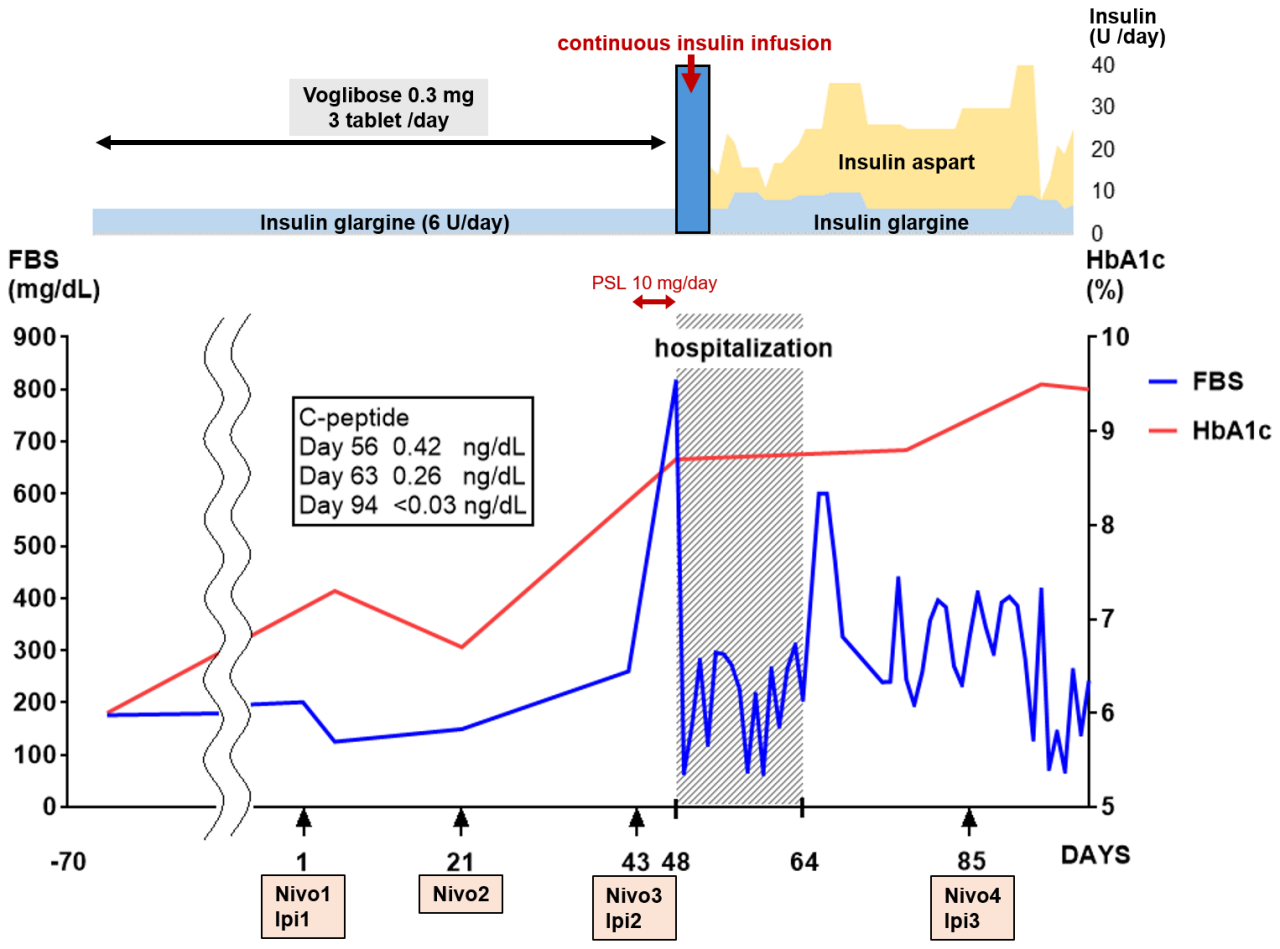
Clinical course. On the 48th day after the start of immunotherapy, the blood glucose level spiked; thereafter, the blood glucose levels fluctuated. The HbA1c and C-peptide levels increased and decreased rapidly, respectively. Continuous intravenous insulin was administered at 0.2 to 5.0 U/h from day 48 to day 50. FBS, fasting blood sugar; HbA1c, glycated hemoglobin; Ipi, ipilimumab; nivolumab, nivolumab.

Following discharge, immunotherapy was resumed on day 85. Unfortunately, tumor growth persisted despite ICI treatment. Therefore, immunotherapy was discontinued six months post-discharge, and chemotherapy with carboplatin and paclitaxel was initiated as the second-line treatment. After completing six courses of treatment, tumor shrinkage was observed, and the patient is still under treatment and follow-up. The clinical course of the patient is summarized in **Figure 2**.

## 3 Discussion

In this report, we describe a case of strong GAD antibody positivity and T1DM-susceptible haplotype DRB1*04:05-DQB1*04: 01 in a Japanese patient with ICI-T1DM. This case presents several notable findings. First, although the prevalence of GAD antibody positivity in ICI-T1DM is lower in Japan than in Western countries, our patient exhibited a high GAD antibody titer, along with a remarkably short duration between the first ICIs and the acute onset of symptoms. Second, we identified the HLA haplotype as DRB1*04:05-DQB1*04:01, susceptible to T1DM haplotypes. Considering these findings, below we have discussed and reviewed the literature on GAD antibody-positive Japanese patients with ICI-T1DM and HLA haplotypes.

### 3.1 A brief review of ICI-T1DM

ICI-T1DM, also known as CIADM, has recently been recognized as a new category of T1DM (6). ICIs may inhibit PD-1 on the surface of T cells or PD-L1 on the surface of tumor cells, thereby activating T cells and enhancing tumor immunity. As PD-L1 is expressed in Langerhans cells, ICIs may block the PD-1 pathway, potentially activating self-reactive T cells to attack Langerhans cells (11, 16).

The global prevalence of ICI-T1DM is approximately 1–1.8% (17–20). ICI-T1DM is characterized by an intermediate onset that is between acute-onset and fulminant T1DM, according to the speed of loss of β cells (21, 22). Therefore, ICI-T1DM may have a different pathogenesis from that of acute-onset or fulminant T1DM. Although race (5), islet antibodies (5, 10–13, 23), and HLA haplotypes (5, 10–13, 24) may be important factors in new-onset ICI-T1DM, their roles have not yet been conclusively determined. We have summarized the characteristics of GAD antibody-positive Japanese patients with ICI-T1DM and their associated HLA haplotypes in **Table 2**.

**Table 2:** Review of anti-GAD antibody-positive Japanese ICI-T1DM cases.

Case	Reference	Age	Sex	Height (cm)	BMI (kg/m^2^)	Type of cancer	Drug	Cycle of ICI treatment^†^	T1DM Onset time (week)	Presence of DKA	Plasma glucose (g/dL)	HbA1c (%)	Serum C-peptide (ng/mL)	GADA (U/mL)	IA–2A (U/mL)	Other islet–associated antibodies	HLA	Other irAEs	History of diabetes	Other features
1	Ishino et al. (25)	65	Male	167.6	21.1	Non–small cell lung cancer	Pembrolizumab	Unknown	8	DKA	757	10.3	2.2	9.7	<0.4	IAA <125 nU/mL ZnT8A <10 U/mL	DRB1*04:05 DQB1*04:01 DPB1*02:01, 05:01	Thyroiditis	SPIDDM (treated as T2DM)	—
2	Inoue et al. (26)	74	Female	157.7	14.2	Non–small cell lung cancer	Atezolizumab	2	23	DKA	739	12.1	0.5	63.2	<0.6	ZnT8A <10 U/mL IAA <0.4 U/mL ICA Negative	DRB1*04:05 DQB1*04:01	—	—	—
3	Enokida et al. (27)	48	Female	155.7	19.3	Salivary gland cancer	Pembrolizumab	5	12	Unknown	317	9	0.45	101	Unknown	Unknown	Unknown	—	—	—
4	Terashi et al. (28)	71	Male	Unknown	Unknown	Small cell lung cancer	Durvalumab	1	4.3	DKA	761	7.2	0.4	48.2	<0.6	Unknown	Unknown	Thyroiditis	—	—
5	Atari et al. (29)	67	Male	169	21.9	Non–small cell lung cancer	Nivolumab	2	5.4	Unknown	343	8.9	0.39	183	Unknown	IAA <125 nU/mL	Unknown	—	SPIDDM (treated as T2DM)	—
6	Yamaguchi et al. (30)	55	Male	175.6	23.4	Renal cell carcinoma	Nivolumab Ipilimumab	1(Nivo) 1(Ipi)	5.4	None	482	9.8	0.44	1160	>30	IAA <125 nU/mL	DRB1*08:03–DQB1*06:01 DRB1*09:01–DQB1*03:03	Thyroiditis	SPIDDM	—
7	Usui et al. (31)	31	Male	Unknown	Unknown	Non–small cell lung cancer	Nivolumab	1	1.9	DKA	743	6.4	<0.03	1760	Unknown	Unknown	DRB1*04:05–DQB1*04:01	—	—	—
8	Matsuura et al. (32)	78	Male	156	23.2	Non–small cell lung cancer	Nivolumab	3	6.1	None	527	6.1	<0.1	41.1	Negative	ZnT8 Negative	DRB1*0301–DQB1*0803 DRB1*0601–DQB1*1406	—	T2DM	—
9	Kawata et al. (33)	84	Male	Unknown	Unknown	Urothelial carcinoma	Pembrolizumab	2	4	Unknown	1124	6.6	<0.061	>2000	1.3	Unknown	DRB1*04:05–DQB1*04:01 DRB1*08:02–DQB1*03:02	—	Unknown	Autopsy Case
10	Kawata et al. (33)	55	Male	Unknown	Unknown	Hodgkin lymphoma	Nivolumab	5	24	Unknown	339	10.1	0.12	6.8	Negative	IAA Negative	A24, A26 B39, B51, DR4, DR14	Thyroiditis	Unknown	Autopsy Case
11	Ishiguro et al. (34)	67	Male	Unknown	Unknown	Malignant melanoma	Nivolumab	4	8.1	None	539	7.1	2.6	>2000	Unknown	Unknown	DRB1*08:02–DQB*03:02	Thyroiditis/ Isolated adrenocorticotropin deficiency	—	—
12	Honoki et al. (35)	66	Male	Unknown	Unknown	Non–small cell lung cancer	Atezolizumab	Unknown	8	None	Unknown	10	Undetectable	44.8	>30	Unknown	DRB1 01:01:01, 09:01:02 DQB1 05:01:01, 03:03:02	—	T2DM	—
13	Fujita et al. (36)	74	Female	Unknown	Unknown	Urothelial carcinoma	Pembrolizumab	Unknown	16	DKA	1119	9.8	0.55	538.5	Unknown	Unknown	DPB1*05:01	Thyroiditis/ Hypophysitis	—	—
14	Urata et al. (37)	76	Male	Unknown	Unknown	Malignant melanoma	Nivolumab	6	12	Unknown	775	8.4	1.1	Positive	Unknown	Unknown	Unknown	Thyroiditis	—	—
15	Our case	66	Male	164	23.1	Non–small cell lung cancer	Nivolumab Ipilimumab	3(Nivo) 2(Ipi)	6.9	DKA	815	8.7	0.42	>2000	< 0.6	IAA < 0.4 U/mL	DRB1*04:05:01, 13:02:01 DQB1*04:01:01, 06:04:01	—	T2DM	Under dialysis

^†^If the time to onset is expressed in months, it is multiplied by 4 and expressed in weeks. If the time to onset is in days, it is divided by 7 to obtain the number of weeks.

BMI, body mass index; DKA, diabetic ketoacidosis; DM, diabetes mellitus; GADA, glutamic acid decarboxylase antibody; HbA1c, glycated hemoglobin; HLA, human leukocyte antigen; IA–2A, insulinoma‐associated antigen–2 antibody; IAA, anti-insulin antibody; ICA, islet cell antibody; ICI, immune checkpoint inhibitor; Ipi, ipilimumab; irAEs, immune-related adverse events; Nivo, nivolumab; SPIDDM, slowly progressive insulin-dependent diabetes mellitus; T1DM, type 1 diabetes mellitus; T2DM, type 2 diabetes mellitus; ZnT8A, zinc transporter 8 antibody.

### 3.2 GAD antibody positivity in acute-onset Japanese ICI-T1DM

In our case, our patient exhibited a high GAD antibody titer with a considerably short symptom onset duration from the first ICIs. The positivity rate of islet-related antibodies in ICI-T1DM is reportedly approximately 26–56%, which is lower than that in T1DM (5, 10–13, 23, 38). Additionally, Qiu et al. reported a positivity rate of 45.7% and 9.5% in islet-related antibodies in Caucasians and Asians with ICI-T1DM, respectively (5). Notably, the positivity rate of islet-related antibodies in ICI-T1DM in Asians is low (5, 9, 24). To further elucidate these characteristics, we searched for Japanese patients with GAD antibody-positive ICI-T1DM.

To the best of our knowledge, so far, 15 cases have been reported, as summarized in **Table 2** (25–37). The mean age of the patients was 65.1. The HbA1c level at the time of ICI-T1DM onset was 8.7%, and the duration of ICI-T1DM onset after the first ICIs was 9.7 weeks. DKA was observed in 6 cases, 3 cases had type 2 DM, and 3 cases had slowly progressive insulin-dependent DM. Baden et al. reported that the mean onset duration from the first ICI was 22.1 weeks in 22 Japanese ICI-T1DM cases, with only one patient testing positive for GAD antibodies (9). Similarly, Wu et al. reported a mean onset duration from the first ICI of 21.5 and 15.6 weeks in islet antibody-negative and -positive ICI-T1DM cases, respectively. Therefore, the onset duration in the reviewed patients with ICI-T1DM, including the current case, was short, consistent with previous reports (5, 10–13, 23). In our current case, the onset duration was 47 days (6.7 weeks), which is considerably short.

The prevalence of DKA is reportedly high in cases positive for islet-related antibodies (10, 13). Our review indicated a DKA prevalence of 38.9%, similar to that reported by Baden et al. (9). The reasons behind the differences reported in the clinical courses between GAD-positive and GAD-negative cases in ICI-T1DM remain unclear. In T1DM, anti-islet autoantibodies are generated as a result of β-cell destruction by T cells (39), and GAD antibody titers do not always reflect disease progression in T1DM (39). However, our results suggest that GAD antibodies may serve as surrogate markers of disease progression in some ICI-T1DM cases.

Predicting new-onset ICI-T1DM is difficult, as demonstrated in our case. In a recent retrospective cohort study, Akturk et al. reported that blood glucose monitoring at every ICI infusion visit failed to detect new-onset ICI-T1DM (40). They proposed that continuous glucose monitoring or daily self-monitoring may be useful for some high-risk patients (40). Although we acknowledge their suggestion, in our case, despite daily self-monitoring, early detection of new-onset ICI-T1DM was not achieved. Initially, we attributed the elevated glucose levels to prednisolone, delaying the recognition of acute glucose elevation and subsequent unconsciousness. Therefore, identifying high-risk patients could facilitate early intervention, such as initiating early insulin injections or increasing the dose, to prevent worsening metabolic disorders related to elevated glucose levels.

The onset of ICI-T1DM is unpredictable, suggesting that monitoring GAD antibody titers could serve as a valuable tool for detecting new-onset ICI-T1DM. However, there are currently no clear criteria for the timing of its measurement. One of the reasons for this is that the pattern of change in GAD antibody titer varies, with some cases reported to have been positive before the start of immunotherapy (41, 42), others to have become positive after the start of immunotherapy (43), and still others to have increased antibody titers after the start of immunotherapy (29, 30). Considering this issue, we propose that anti-GAD antibodies should be measured before immunotherapy, and positive cases should be considered high-risk patients and periodically monitored after the start of immunotherapy to confirm that the titer does not increase rapidly. Another issue is that this assessment plan cannot be applied to cases that become positive for GAD antibodies after the start of immunotherapy. As mentioned above, the average period from the start of immunotherapy to the onset of disease in Japanese ICI-T1DM patients with positive GAD antibodies is 9.7 weeks (n=15). In this regard, measuring GAD antibodies once, for example, a few weeks after the start of immunotherapy, may be useful. While it is difficult to suggest a timing for GAD antibody measurement at present, this is a clinically important issue, and further studies are needed in this regard.

In addition, although islet antigen-2 (IA-2) and zinc transporter 8 (ZnT8) antibodies have been reported to be surrogate markers of pancreatic beta cell destruction (39), their titers were not high in our review (**Table 2**). Wu et al. reported that multiple autoantibodies are less frequently detected in ICI-T1DM than in T1DM (13), possibly indicating low positivity rates for IA-2 and ZnT8 antibodies in patients with ICI-T1DM. Therefore, anti-islet antibodies, such as GAD or IA-2 antibodies, may play different roles in T1DM and ICI-T1DM, and future studies should focus on elucidating their roles.

### 3.3 T1DM-susceptible haplotype DRB1*04:05-DQB1*04:01 in our patient

The association between HLA haplotypes and ICI-T1DM remains unclear. However, the HLA haplotype of our patient was identified as DRB1*04:05-DQB1*04:01, a T1DM-susceptible haplotype.

First, the association between HLA haplotypes and T1DM and the frequency of HLA haplotypes vary across racial and ethnic groups (44). For example, DR3 and DR4 are important susceptible HLA haplotypes in Europe and Africa. However, in Japan, DR3 is rare, and instead, DR4 and DR9 are important susceptible HLA haplotypes (45). Additionally, susceptible HLA haplotypes vary by race, even in East Asia. For example, DR4, DR9, and DR3 HLA haplotypes have been reported in Korea (46) but not in Japan. Moreover, acute-onset and fulminant T1DM have different HLA-susceptible haplotypes. Although DRB1*04:05-DQB1*04:01, DRB1*08:02-DQB1*03:02, and DRB1*09:01-DQB1*03:03 are typically detected in Japanese patients with acute-onset T1DM, DRB1*08:02-DQB1*03:02 is not associated with the HLA haplotype of fulminant T1DM (15). Therefore, if ICI-T1DM is categorized as a new T1DM subtype, ICI-T1DM-related HLA haplotypes may differ from those observed in T1DM. Some patients with ICI-T1DM exhibit a T1DM-resistant HLA haplotype (13, 24). In a study conducted in Japan, Inaba et al. reported a high frequency of HLA-DRB1*04:05-DQB1*04:01-DPB1*05:01 or DPA1*02:02-DPB1*05:01 haplotypes in ICI-T1DM (47, 48). In our case, the T1DM-susceptible haplotype-DRB1*04:05-DQB1*04:01 was detected, which may also be associated with the HLA haplotype of Japanese ICI-T1DM.

Second, an association between GAD antibody positivity and HLA haplotypes has been reported. Tsutsumi et al. reported that although both DRB1*04:05-DQB1*04:01 and DRB1*09:01-DQB1*03:03 are frequent in Japanese fulminant T1DM, GAD antibody-positive fulminant T1DM was only significantly associated with DRB1*09:01-DQB1*03:03, even with considerably low GAD antibody positivity rates (14). As shown in **Table 2**, DRB1*04:05-DQB1*04:01 has been detected in five cases, including ours (25, 26, 31, 33). Yamaguchi et al. reported DRB1*09:01‐DQB1*03:03 in a patient (30). In contrast, Imagawa et al. reported a high frequency of DR9 in Japanese ICI-T1DM cases (49), which is inconsistent with the observations from our review. These findings suggest that susceptible HLA-related haplotypes differ in Japanese patients with ICI-T1DM positive for GAD antibodies, warranting further studies with larger sample sizes.

### 3.4 Strengths and limitations

In this study, we reviewed Japanese patients with GAD antibody-positive ICI-T1DM and their associated HLA haplotypes. Our study highlights several important findings. First, GAD antibody positivity may be associated with acute onset and disease progression in some cases of Japanese patients with ICI-T1DM. Second, as the prediction of new-onset ICI-T1DM is challenging, monitoring GAD antibody levels might be useful. Third, DRB1*04:05-DQB1*04:01 is a potentially important HLA haplotype of GAD antibody-positive ICI-T1DM in Japanese patients. These findings provide important insights into guiding clinicians in treating patients undergoing ICI therapy.

This study has several strengths. First, as only a few cases of GAD antibody-positive Japanese ICI-DM have been reported, the characteristics of this condition remain unclear. To the best of our knowledge, this is the first review focusing on GAD antibody-positive Japanese patients with ICI-T1DM. Recently, various rare onsets and related cases of ICI-related T1DM have been reported (50–52), and our study adds valuable inputs for understanding the pathogenesis and management of this condition. Second, we have discussed the HLA haplotype of GAD-positive antibodies in a small number of Japanese ICI-T1DM cases, warranting future investigations with larger sample sizes and validation across different racial and ethnic groups.

However, this study has some limitations. First, we could not measure GAD antibody titers before ICI treatment. Second, data on islet–associated autoantibodies other than GAD antibodies are limited. Third, we could not determine the HLA haplotypes of DPA1 and DPB1 owing to phase ambiguity. Fourth, although we carefully searched the literature, some reports did not contain detailed information, and we could not draw detailed clinical manifestations in some cases. Finally, no individual GAD-positive cases were identified in several observational studies.

## 4 Conclusions

In summary, we present a case of strongly positive GAD antibody and T1DM-susceptible haplotype DRB1*04:05-DQB1*04:01 detected in a Japanese patient with ICI-T1DM. As the high-GAD antibody titer ICI-T1DM may be acute and have a short onset from the first ICIs, clinicians should carefully observe patients. However, our results should be validated in studies with large sample sizes and across different racial and ethnic groups.

## Data availability statement

The raw data supporting the conclusions of this article will be made available by the authors, without undue reservation.

## Ethics statement

The studies involving humans were approved by the Shirakawa Kosei General Hospital Ethics Committee-Decision No HAKURIN23-011, June 19, 2023. The studies were conducted in accordance with the local legislation and institutional requirements. The participants provided their written informed consent to participate in this study. Written informed consent was obtained from the individual(s) for the publication of any potentially identifiable images or data included in this article.

## Author contributions

SY: Conceptualization, Data curation, Formal analysis, Investigation, Methodology, Writing – original draft, Writing – review & editing. HH: Conceptualization, Data curation, Formal analysis, Investigation, Methodology, Writing – original draft, Writing – review & editing, Supervision. CM: Data curation, Investigation, Writing – review & editing. YK: Supervision, Writing – review & editing. TH: Supervision, Writing – review & editing.
